# “I Am Not the Same as I Was Before”: A Qualitative Analysis of COVID-19 Survivors

**DOI:** 10.1007/s12529-022-10129-y

**Published:** 2022-10-13

**Authors:** Emily Duan, Kira Garry, Leora I. Horwitz, Himali Weerahandi

**Affiliations:** 1 NYU Grossman School of Medicine, New York, NY USA; 2 University Park Program, Pennsylvania State College of Medicine, State College, Hershey, PA USA; 3 Division of Healthcare Delivery Science, Department of Population Health, NYU Grossman School of Medicine, New York, NY USA; 4 Center for Healthcare Innovation and Delivery Science, NYU Langone Health, New York, NY USA; 5 Division of General Internal Medicine and Clinical Innovation, Department of Medicine, NYU Grossman School of Medicine, 227 East 30th St., NY 10016 New York, USA

**Keywords:** Chronic COVID-19, Long COVID, Post-acute COVID-19, Qualitative study

## Abstract

**Background:**

Little is known about the illness experience of patients’ long-term emotional and physical recovery from severe COVID-19 infection. This study aimed to expand upon the recovery process of COVID-19 survivors up to 6 months after hospital discharge.

**Methods:**

Qualitative analysis of free-response answers from a cohort study of 152 patients ≥ 18 years hospitalized with laboratory-confirmed SARS-CoV-2 surveyed at 1-month post hospital discharge and 6-months post hospital discharge. Responses were analyzed with a grounded theory approach to identify overarching themes.

**Results:**

Participants described persistent complications, both physical and mental, that have affected their recovery from COVID-19. Five overarching themes of post-acute patient experiences were generated: (1) an increased awareness of a mind and body connection, (2) feelings of premature aging, (3) an overall decline in quality of life, (4) a continued fear of infection, and (5) methods of coping.

**Conclusions:**

Patients described lasting changes to their mental health and overall quality of life in connection to physical complications after severe COVID-19 infection. Patients’ reports of their experience call for a greater awareness of the psychological aspects of COVID-19 recovery to provide both physical and psychological rehabilitation services. Additional resources such as education around re-infection and financial resources are needed.

## Introduction

In the USA, over 90 million patients have been infected with COVID-19 with over 4 million patients requiring hospital admission [1]. Some patients in recovery are now describing prolonged symptoms of fatigue, post-exertional exhaustion, and cognitive dysfunction even months out from diagnosis [2–5]. International studies examining these “COVID long haulers” have primarily focused on the physiologic complications associated with COVID-19 infection, such as respiratory symptoms, thrombosis, and cardiovascular complications [6].

These findings are expected, as many patients hospitalized with severe COVID-19 infection have faced significant complications like acute respiratory distress syndrome (ARDS) and long intensive care unit stays. Along with physical symptoms, many patients recovering from COVID-19 struggle with persistent mental health issues and a decline in quality of life [7, 8]. Prior studies of post-ICU patients demonstrate post-traumatic stress disorder (PTSD)–like symptoms and fatigue persist for months to years after hospital discharge, with an estimated prevalence of PTSD-like symptoms as high as 62% [9, 10]. Likewise, research on prior coronavirus outbreaks such as the severe acute respiratory syndrome (SARS) and the Middle East respiratory syndrome (MERS) has recognized the persistent psychological distress that survivors of epidemics face, including elevated levels of stress, anxiety, and depression [11–14]. Similar findings are beginning to emerge in patients with COVID-19 [15–17]. More recent studies on long COVID have revealed that over half of patients in recovery experience fatigue at 10 weeks after COVID-19 infection [18]. Other symptoms like difficulty concentrating, anxiety, depression, and cognitive blunting are common in patients with long COVID [19, 20].

To better understand the ramifications of these new chronic symptoms on patients’ daily lives, a deeper exploration of the individual experience of the recovery process is necessary. Personal accounts of long COVID and patient-focused qualitative studies have become more widespread, providing valuable insight into the experience of COVID-19 recovery [21–23]. Early qualitative literature from the pandemic coming predominantly from China, but also some from the USA, focused mostly on healthcare providers’ and caregivers’ experiences [24–28]. These studies highlight the emotional burden of caregivers managing patients with severe disease. Several of the early studies on patient recovery were conducted outside the USA (Iran, China, Italy) or focused on specific ethnic groups [29–32]. The impact of COVID-19 in the USA is interesting, because it has exposed structural factors that predispose certain racial and ethnic minority groups, such as African American, Native American, and LatinX communities, to greater morbidity and mortality from COVID-19 infection [33]. In particular, New York City was the epicenter of the US pandemic, and was first to be hit with a surge in critically ill patients, imposing great distress on healthcare resources and underprepared medical institutions and exposing societal disparities. More recent qualitative studies focus on populations identified from social media or online support groups [21, 34]. One systematic review highlighting five of the existing qualitative studies describes common identified analytical themes around symptoms of long COVID, self-directed management, and the emotional aspects of living with long COVID [34, 35]. They also highlight the need for more qualitative research on culturally diverse samples of people living with long COVID to better understand the impact and to inform rehabilitation strategies.

Further research is needed to provide a broader understanding of the nuances of surviving severe COVID-19 and the long-term consequences of living through a pandemic. Therefore, we sought to investigate the lived experience of a cohort of COVID-19 survivors in a US, New York–based population by examining their open-ended responses to a survey about life after hospital discharge. We utilized qualitative analysis to explore patients’ experience of their physiologic and emotional recovery.

## Methods

### Study Settings and Participants

This study was a qualitative analysis of data collected from a previously described single health system observational cohort study in New York, USA, and was approved by the Institutional Review Board at NYU Grossman School of Medicine [36, 37]. Recruited participants came from NYU’s Tisch Hospital in Manhattan, NY; NYU Brooklyn Hospital; and Winthrop Hospital in Long Island, NY; these hospitals serve patients in the Greater New York area. Methods used are described below and follow the COREQ and SPQR recommendations for conducting qualitative research [38–41].

This cohort study followed patients ≥ 18 years hospitalized in one of the three New York NYU hospitals (Manhattan, Brooklyn, Long Island) with laboratory-confirmed SARS-CoV-2 who required at least 6 L (L) of oxygen during their admission, had intact baseline cognitive and functional status, and were discharged alive after April 15, 2020. At approximately 1 month post-discharge and 6 months post-discharge, patients were invited to participate in an online or telephone survey about their recovery from COVID-19. Enrollment, including a detailed flow diagram of excluded, eligible, and enrolled participants, is detailed in our 1-month outcomes paper [37]. Our exclusion criteria included patients with communication impairment or baseline dementia which was determined by chart review or if, upon consent for the study, the patient was unable to articulate the purpose of this study and what would be required of them to participate. We also excluded patients discharged to hospice, patients who resided in long-term-care pre-hospitalization, patients fully dependent on activities of daily living pre-hospitalization, and patients that opted out of research. Demographics for the full cohort included age; sex; language and, if used, interpreter; race; health insurance; smoking status; and other health demographics based on COVID hospitalization. Our 1-month and 6-month outcomes papers include these full demographic tables [36, 37].

The last question of the 1-month and 6-month surveys was a free-response question that asked: “Is there anything else you would like to tell us about how your life has been since being discharged from the hospital?” If patients opted to complete the online version of the survey, patients typed their answer in a 65,000-character-limit textbox. If completed by phone, research staff interviewers transcribed responses verbatim to the best of their ability. Interviewers asked clarifying questions and continuing statements such as “Can you tell me more about your fatigue post-COVID?” Participants were invited to speak as much as possible about their experience.

### Data Analysis

Data was collected on the secure platform REDCap and analyzed in Excel spreadsheets. The free-response answers were analyzed by inductive coding, following a grounded theory approach with subsequent iteration and constant comparison. In analyzing across the time frame, we found no differences in codes or themes. Therefore, the 1-month and 6-month answers were grouped together during coding. Four investigators (KG, ED, LH, and HW) first briefly reviewed 134 free-response answers as a group to develop a preliminary codebook. The preliminary coding schema totaled 29 codes. Then, KG and ED double coded a set of 20 responses using the preliminary codebook and compared codes. Any discrepancies were reviewed and reconciled with LH and HW via virtual team meetings, and subsequent adjustments to the codebook were made. KG and ED then moved onto the next set of 20 responses. This iterative process was undergone 6 times, until the data set was coded completely. KG and ED reached thematic saturation during the 5th set of coding. After all responses were coded, the final codebook was then used to complete a re-coding of all 134 responses. The codes were then consolidated into 5 categories by consensus. Categories were iteratively consolidated into emerging themes through team discussion.

## Results

### Description of the Dataset

Of 152 patients in the overall survey cohort, a total of 134 participants (87.5%) responded to the free-response question at either the 1-month survey (34.3%), 6-month survey (8.2%), or both (57.4%). Overall, 115 (85.8%) participants answered the free-response answer by phone, 16 (11.9%) participants typed their response in the online survey, and 3 (2.2%) used both methods (phone at 1 month post-discharge and online 6 months post-discharge). The average length of response was 340 characters for phone interviews and 270 characters for online free-text responses. Free-response participants included 81 men (60.4%) with a mean age of 59 years, range 20–91 (see Table 1). The study had 103 participants who listed English as their primary language in the electronic health record (76.9%), 24 participants (17.9%) who listed Spanish, three participants who spoke Russian, two who spoke Bengali, one who spoke Cantonese and one who spoke Mandarin. Of the 134 total participants, 25 (18.7%) completed the telephone interview using an interpreter, and all translated responses were included in the analysis. In the full cohort, in the 1-month survey, the population (*n* = 145) included 69 (45%) non-Hispanic White, 32 (21%) Hispanic, 16 (11%) other/multiracial, 16 (11%) Asian, 13 (9%) non-Hispanic Black, and 6 (4%) unknown. In terms of health insurance, 60 (39%) had commercial insurance, 52 (34%) Medicare, 36 (23%) Medicaid, and 4 (3%) unknown. The full data of race, health insurance, and other demographics are in our 6-month outcomes paper [36].

**Table 1:** Study cohort characteristics

**Study cohort characteristics**	
**Study characteristic**	***n* (%) or mean (range)**
***Gender***	
Male	81 (60.4%)
Female	53 (39.6%)
***Age (years)***	59 (20–91)
***Language***	
English	103 (76.9%)
Spanish	24 (17.9%)
Russian	3 (2.2%)
Bengali	2 (1.5%)
Chinese–Cantonese	1 (0.7%)
Chinese–Mandarin	1 (0.7%)
***Used interpreter***	25 (18.7%)
**Total participants with free-response answers**	**134**

### Key Themes

In our qualitative analysis, we revealed 5 overall themes related to the physiologic, emotional, and psychosocial impacts of COVID-19 on survivors: (1) mind–body connection, (2) shifts in identity, (3) COVID-19’s impact on quality of life, (4) fear of infection, and (5) methods of coping (see Table 2). Within the five main themes, there were 21 total sub-codes applied to a total of 361 phrases or sentences.

**Table 2:** Summary of themes identified as COVID-19-related factors affecting patient’s lives

**Key themes identified from patients recovering from COVID-19**	**Representative quotes**	**Number of times the code was applied**
**Mind–body connection**		
Distrust of one’s body	*“It’s going to take a while for me to learn how to do what I used to be able to do without thinking. I’m trying to be careful not to exert myself too much”*	10
New uncertainty of everyday behavior	*“I felt completely depleted of my ability to do day-to-day things."* *“I feel a little tired. I’m not the same as before…I cannot walk the same as before. I do not know if they will ever be the same. I cannot walk the way I used to.”*	28
**Shifts in identity**		
Feeling that COVID-19 has accelerated the aging process	*“I am 77, but I never thought of myself as old. I work full time, I walk everywhere, I do my own housework and gardening. Somehow, despite the fact that I can do all of those things again, I sometimes mentally feel like I am an old lady, and it bothers me a lot. It has never been like this before, and it is frustrating.”*	6
Loss of independence	*“I’ve hired additional aides to help with things like cleaning and laundry. It’s felt very confining being stuck at home.”* *“My husband is a great help and takes care of a lot of things in the house. I sometimes feel uncomfortable. I have to depend on other people to go to a store and come back to the house.”*	11
**COVID’s impact on quality of life**		
** Emotional**		
Low mood	*“Health wise, I have recovered well. But mentally, not seeing much of a difference. I am still sad and depressed."*	7
Anxiety	*“I am now on anxiety meds as a result, which is something I haven’t had to do in 10 years…I don’t think a lot of people realize that. a lot of people are going undiagnosed with anxiety… mental health is part of the healing.”*	14
Isolation	*“Sometimes I get bored, but there’s nowhere to go since everything is closed, so socially it hasn’t been easy either. Slowly I’m trying to get back to normal.”*	11
** Impact on home and work**		
Employment difficulties	*“Because of coronavirus, I cannot work anymore, so I cannot pay my rent. They evicted me, and I could not even get my clothes. For two or 3 days, I was living on the street. Because the pandemic is still going on, I cannot work and I am weak. I am not educated so I cannot get another job.”*	7
Financial difficulties	*“I’m trying to get unemployment [benefits] but I still haven’t heard from anyone yet"* *“I still get tired easily…I get depressed sometimes when I think about my hospital bills"*	4
Impact on family	*“I was gone for three months, and my family had to take care of the house. My oldest son was trying to finish up college and he was watching my younger son, and he had to take care of the bills including the mortgage.”*	2
** Physical**		
Physical complications	*“When I was discharged, I had 2 weeks of tremendous stomach pain and gas, the doctor treated it, thank god it’s gone.”* *“I have slight upper body weakness. In terms of upper body ache, get some numbness across the shoulders. I got some numbness across my face”*	82
Feeling like life is not the same	*“I am not the same as I was before. I am much weaker, completely different, and not as active.”*	10
Feelings about a prolonged recovery	*“I think I will just need more time to fully recover.”* *“Sometimes I still feel like I have residue of the disease”*	17
** Methods of coping**		
Change in outlook	*“I find myself being thankful, mostly. I’d like to think I always was. But I feel a greater sense of peace and appreciation for life after going through what I went through. Both intellectual understanding of the physical duress and recovery.”*	8
Gratitude	*“My life has been great. I just feel so lucky, especially not to have any lingering effects. Glad to be back to normal. I am feeling very lucky.”*	65
Healing process	*“I’m trying to heal my body as much as possible. I go for daily walks. I started bike riding in the past week or so, and have been able to go about 3 times this week. I’ve been really pushing myself to exercise even when I don’t feel like it.”*	20
COVID as turning point for change in health	*“I am forced to look at things differently and take my health more seriously. I was really sick because of the choices I made before and I had a heart attack a couple years ago. I was told I needed to lose weight, but didn’t. Then I got COVID and was in the hospital for almost a month and that really changed my perspective. I want to get better and I want to do better. Now, I make time to take care of my health.”*	15
Social and community support	*“It’s been excellent, because my husband has stepped up. My daughter is also here, so between the two of them, I’m set.”* *“I normally live alone, but I’ve been living with my sister since getting out of rehab.”*	11
** Fear of infection**		
Anxiety about the future	*“It’s kind of stressful to not know what the future is. With my underlying condition, I am afraid to socialize and get back to work, since I work in a school system. So I don’t know what to do.”*	3
Feelings of uncertainty about recovery	*“The recovery process has been long, not only weaning off the oxygen but getting back to a place where I can go back to everyday activities…Not knowing if there are long term effects is something that worries me.”*	15
Risk of contagion	*“I’ve been very careful and cautious because I really don’t want to get coronavirus again. I try to avoid going out and I attend church services over the phone.”*	15

#### Theme 1: Mind–Body Connection

Participants expressed an explicit connection between their physical recovery and their mind. In particular, some patients described a distrust of their body, explaining that although they had a desire to do something physically, they felt uncertain and anxious whether their body could handle it after COVID-19: *“It’s going to take a while for me to learn how to do what I used to be able to do without thinking…. I’m getting used to feeling normal* [Patient ID 126].*”* Participants described their body as a “trigger” and experiencing a PTSD-like stress response when trying to engage in activities that were difficult during their acute COVID-19 illness: *“When [I] first started to get sick, [I] became very weak and had to hold the walls when [I] showered. [I no] longer have weakness, [but] now experience anxiety [and] possibly panic attacks when [I] shower* [Patient ID 2].*”*

Participants also explained new uncertainty in undertaking their prior everyday behaviors, including walking, cooking, cleaning, and washing laundry, which made them doubt their body’s capacity. One participant said, *“I’m not the same as before. My legs get tired. I cannot walk the same as before. I do not know if they will ever be the same.”* and another, *“I felt completely depleted of my ability to do day-to-day things* [Patient ID 20].*”*

#### Theme 2: Identity Shifts: Accelerated Aging and Loss of Independence

Several patients reported a change in their perception of self, related to dramatic changes in lifestyle. For example, patients reported feeling like COVID-19 had aged them. This included an awareness of physical symptoms typically associated with aging such as feeling slower, increasing forgetfulness, and disrupted sleep patterns. For others, there was a sense of “differentness,” of transforming into an older version of oneself independent of any functional changes from COVID-19 complications. Even patients who reported minimal changes to their daily activities of living throughout the recovery process noted that they now perceive their identity as an “old person.” One patient described, *“I am 77, but I never thought of myself as old. I work full time, I walk everywhere, I do my own housework and gardening. Somehow, despite the fact that I can do all of those things again, I sometimes mentally feel like I am an old lady, and it bothers me a lot* [Patient ID 77]*.”*

Another core feature of patients’ shifts in identity was feelings of frustration about their loss of independence. Causes for decreased independence included shortness of breath, dependence on oxygen, fatigue, and weakness. One patient said, “*[I]t has felt very confining being stuck at home [due to my shortness of breath]* [Patient ID 95].*”* As a result of these complications, patients reported an increased reliance on friends, family, and home health aides to assist in activities of daily living. Survivors frequently mentioned their frustration with this new dependence on others and inability to engage in hobbies and extracurricular activities: *“I’m a very happy and positive person, but a lot of things have stopped: I cannot do anything that I used to. I cannot clean the house, I cannot go outside and garden. Even if there were no restrictions, I don’t think I’d be able to go about my usual activities* [Patient ID 96].*”*

#### Theme 3: COVID-19’s Impact on Quality of Life

The most prevalent theme found in these free responses was the impact the virus had on patients’ quality of life. Sub-themes included physical impacts (e.g., physical complications, experiences related to prolonged recovery), emotional impacts (e.g., low mood, isolation, and anxiety), and impacts on work/home (e.g., employment difficulties, financial difficulties, and impact on family).

- Physical Impacts
  By far, the most common impact on quality of life was persistence of physical symptoms following COVID-19 infection. We found that, amongst the responders to the free-response question, over half of the participants spoke about their physical complications. The most mentioned complications included fatigue, weakness, shortness of breath, and both neuropathic and musculoskeletal pain. In some cases, patients spoke more globally about the impact of COVID-19 on their overall state of health or life. We found an overarching theme of “feeling that life is not the same.” One patient said, *“It completely changed my life. I am not the same person as I was before.* [Patient ID 90].*”* Additionally, patients often mentioned a feeling that they “just needed more time” to recover or even offered advice about a prolonged recovery: *“I think most of the patients that are recovering should really ingrain in them to have patience.* [Patient ID 43].*”* One patient said, *“Everybody just needs space and time to get better* [Patient ID 10].*”*
- Emotional Impacts
  COVID-19 also impacted patients’ emotional state. Some patients with anxiety diagnoses prior to COVID-19 reported needing to resume medication after their hospitalization, while others experienced new anxiety around the future of their recovery or the state of COVID-19 in the community: *“I can’t listen when they talk on the news about it, then I get anxiety. I have to get up and leave the room…I have to work through it. I’m speaking with a therapist and my insurance company has somebody I can speak to* [Patient ID 36].*”*
- Impacts on Work/Home
  We found that COVID-19 has had a lasting impact on patients’ lives at work and at home. The median hospital length of stay for the overall cohort was 18 days (interquartile range 10–31), and was an indicator of time that patients had to take off work. Participants also reported challenges of remaining employed due to persistent physical symptoms or the lack of employment opportunities during the pandemic. One patient said, “*My weakness prevents me from delivering food, which is what I normally do, I do not know what to do* [Patient ID 41],*”* and another said, *“I lost 2 of my jobs…I used to be a caregiver and the man died of coronavirus…I’m trying to get unemployment [benefits] but I haven’t heard from anyone yet.* [Patient ID 60].*”* Participants also reported subsequent financial difficulties, due to the lack of employment. For example, a patient said, *“I cannot work anymore, so I cannot pay my rent. They evicted me.* [Patient ID 41].*”*

#### Theme 4: Fear of Infection

Many patients endorsed feelings of anxiety and uncertainty about the future related to the COVID-19 pandemic. For some patients, the primary fear was the possibility of re-infection, *“Everybody is just nervous and if I sneeze my kids are looking at me like ‘Are you getting sick again?’ It’s just very mind boggling. I’m really really worried* [Patient ID 36].*”* Another patient replied, *“God forbid I catch this again, I was close to death, I don’t want to take this risk again.* [Patient ID 34].*”* For other patients, the concern was primarily with the risk of transmission to others and frustration with unclear guidelines on post-discharge quarantine. One patient questioned, *“the [doctors] said I don’t need to be quarantined from my family or wear a mask…this is untrue because my two kids, five and one and a half years old, both have a light inflammatory rash…[and] perhaps I caused that* [Patient ID 3].*”*

Patients also reported a fear of stigmatization of their COVID-19 infection. Patients lamented that it was hard to hear that people *“did not want to be around [me] if they know [I] had coronavirus* [Patient ID 70]*.”* Another mentioned, *“It’s been bad. It’s been a problem for all the people that have been infected with coronavirus that other people despise them because they are afraid they are going to infect them.* [Patient ID 23].*”*

#### Theme 5: Methods of Coping

All patients in our study required inpatient hospitalization and were on at least 6 L of oxygen during admission. Given the severe nature of our patients’ infections, one of the most salient themes that emerged was the coping methods that patients employed throughout their hospitalization and recovery process. Sub-themes included gratitude, social and community support, what the healing process looked like, changes in outlook, and COVID-19 as a turning point for change.

- Gratitude
  A common theme mentioned by patients was a feeling of thankfulness for surviving the COVID-19 virus and a new appreciation for life. These mentions were split into two categories of general gratitude for life and an appreciation for the hospital workers who had taken care of them. One patient mentioned, *“they took such good care of me at [redacted], like I was their family. God bless those people.* [Patient ID 127]*.”* Other patients reported feeling grateful for a second chance, an opportunity to change their life for the better. *“I’m hoping that a lot of the things I’m doing now will help my health and my quality of life. I’m very happy that I survived to recognize that I needed to change, and I’m inspired to keep this momentum moving forward* [Patient ID 109]*.”*
- Social and Community Support
  Several patients recognized relying on their family and community members for support as a part of their recovery. *“After those first 3 weeks, I can remember that the nurses and aides were very good to me. Now that I'm back home, my husband is here and my children come in to help me so I feel very grateful and thank God* [Patient ID 125]*.”*
- Healing Process
  Several patients discussed what their path to both spiritual and physical recovery looked like. In particular, patients relied on physical therapy for both physical recovery from complications but also for management of mood symptoms. One patient mentioned, *“[my] shortness of breath makes me nervous and irritated… so [I am] trying a lot of breathing and physical activities to try to improve [my] breathing* [Patient ID 140]*.”* For others, the healing process involved finding ways to cope with stress, developing healthier eating and exercise habits, engaging in hobbies and mental health therapy, and avoiding negative stressors—*“I walk and exercise. I do all of the strengthening exercises that my physical therapist recommends every day. I actually look at nutrition labels and I’m more careful about what I eat.”* [Patient ID 100]*.”*
- Change in Outlook
  Patients in the study also reported a new outlook on life following their COVID-19 hospitalization and recovery process. Interestingly, several patients felt more positive about their lives and described their COVID-19 hospitalization as a transformative experience. One patient reported, *“I can only say that this experience has brought to me more positivity, greater awareness to being able to help others around me…it’s really brought a remarkable change in my life and in my career as a therapist and as a musician. I use my music to help me. This definitely made me a better person.”* Other patients consistently brought up a theme of “lessons learned from COVID-19 [Patient ID 68],” including having patience for the recovery process and newfound gratefulness for life.
- COVID-19 as a Turning Point for Change
  One of the most surprising themes in this study was a feeling that the COVID-19 infection was a turning point for positive change. For some patients, the positive change was due to improvements in prior health conditions through the physical therapy, rest, and attentive medical care provided during their COVID-19 infections. For one patient, *“[my] health is better now than before being hospitalized because [I] had many pre-existing problems with [my] heart and lungs which the doctors at the hospital were able to improve* [Patient ID 82]*.”* Patients who felt that COVID-19 had improved aspects of their lives also cited improved emotional connection to family and friends as a reason: *“I’ve had a lot of people reach out, and in some ways it’s been the best moment of my life. It brought my family together and a community of friends together* [Patient ID 137]*.”*

## Discussion

This qualitative study explored the recovery experience of patients diagnosed with severe COVID-19 and serves to enrich our quantitative understanding of post-discharge outcomes. Our findings confirm that patients who experience severe COVID-19 infection have lasting physical and psychological symptoms. Through our qualitative analysis, we uncovered that our cohort experienced an alteration in their perceptions of their bodies, characterized by an increased awareness of physical weaknesses and newfound uncertainty about their body’s limits. This distrust of one’s body included a startle response when attempting to re-engage in everyday activities and avoidance of tasks that were difficult during active COVID-19 infection. The avoidant behavior and overwhelming anxiety are reminiscent of the early signs of an acute stress response and PTSD in patients recovering from ICU-level hospitalization [42–44]. Our study adds to the current understanding of the neurocognitive post-acute sequelae of COVID-19, and suggests that longstanding psychological rehabilitation will be key in the recovery process [45–47].

Patients’ enhanced perception of the mind–body connection can also be related to feelings of accelerated aging and shifts in identity during the recovery process. To our knowledge, our paper is the first to describe the phenomenon of “accelerated aging” in recovering patients. Given the disproportionate impact of COVID-19 on the elderly population, our findings add to the current understanding of the psychological toll associated with patients’ loss of independence during the recovery process [48]. Likewise, our patients’ loss of independence sheds light on the importance of physical rehabilitation and ADL assistance through home health aides or other supports in order to be able to return and live at home safely.

Our study encompasses multiple hospitals across urban and suburban settings, and our patient population was markedly diverse. In our full cohort, over half of the participants (52%) identified as a minority group, and 23% had Medicaid and 34% had Medicare as their primary health insurance. Immigrant communities in New York City were hit particularly hard with COVID-19, and our study included patients from diverse backgrounds, with no language exclusions: nearly one in five patients in our study completed the survey through an interpreter. Our participants emphasized the impact on their life outside of the physical complications—they discussed financial difficulties including employment, housing, and hospital bills. While the experience of persistent physical symptoms of COVID-19 has been described both in social media and in recent Post-Acute Sequelae COVID-19 (PASC) papers [49–52], our study adds to this body of work by providing a deeper understanding of the emotional, financial, and other life challenges faced by those who experienced severe COVID-19 infection. One study by Schiavi et al. highlights and corroborates our findings [32]. This Italian qualitative study on the experience of long COVID found that patients narrated the persistence of symptoms described in PASC, especially a sense of isolation and psychological distress. Several of their identified themes match ours such as fear and stigma, isolation, persistent symptoms, and returning to adapted life. As identified by Shiavi et al., our study too found the emotional distress and avoidance of activities such as news or television on COVID-19 may be a sign of PTSD. The predominance of quotes on persistent symptoms was corroborated here, and the quantitative data collected by the REACT study by Fugazzaro et al. and Schiavi et al. adds depth to our understanding of the “vicious circle” of isolation, stigma, and emotional distress in post-COVID patients [19, 32]. As interventions and policies are developed to support patients suffering from PASC, it is important they incorporate screening for acute stress disorder, PTSD, and social determinants of health such as housing security, employment, and financial security.

## Limitations

With the emergence of new data on long COVID, our study sought to unpack the nuances of patients’ long-term emotional and physical recovery. Because this paper was designed to supplement quantitative research on COVID-19-related symptoms and complications, qualitative data was gathered from a free-response section and was not based on structured or semi-structured in-depth interviews. Although our sample size is appropriate for a grounded theory analysis, we did not conduct subgroup analyses because there was an insufficient number of participants to do so. We interviewed patients with more severe infections–those who required hospitalization and at least 6 L of oxygen during admission; as a result, poor health outcomes and a prolonged recovery process may be more prevalent in our study compared to the general population. We recognize that this study’s population was limited to New York (specifically Manhattan, Brooklyn, and Long Island) in the USA and does not fully capture the scope of COVID-19’s impact globally.

## Conclusions

In conclusion, in this qualitative analysis examining post-acute COVID-19 patients’ lives, we identified five overarching themes characterizing their recovery: (1) an increased awareness of a mind and body connection, (2) feelings of premature aging, (3) an overall decline in quality of life, (4) a continued fear of infection, and (5) methods of coping. The lived experiences we have uncovered—their worries, their financial difficulties, and the shifts in their identity—should guide patient care as they continue to recover from the post-acute sequelae of this illness. We found the most important finding of our study to be the described impact of COVID-19 on patients’ overall quality of life. Even 6 months into recovery, patients experienced physical, emotional, and financial ramifications that altered their daily life—some patients remained unable to work, some were anxious over continued hospital bills, and others detailed newly diagnosed anxiety. Our findings show that not only are patients dealing with physical symptoms and rehabilitation, they also are experiencing the all-encompassing nature of feeling that “I am not the same as I was before.” These findings indicate the pervasive effects of COVID-19 infection, and may aid our ability to improve patients’ mental health, long-hauler symptoms, and socioeconomic struggles.

The major themes identified, in combination with the known prevalence of persistent physical symptoms of long haulers, underscore the importance of a multidisciplinary approach in caring for survivors of severe COVID-19 who may deal with problems ranging from premature aging, PTSD-like symptoms, anxiety, social stigmatization, and isolation. Furthermore, this work demonstrates the impact post-acute COVID-19 has had on patients’ social determinants of health. Social work and case management should be integrated into settings caring for these patients to address loss of income from layoffs and housing insecurity. Our patients’ recurrent fears of becoming re-infected with COVID-19 and worries over transmission to family members emphasize a need for the provision of safe quarantine spaces and clear recommendations on household isolation practices.
